# Morphological Analysis of Fractures of the Proximal Humerus by the Fracture Mapping Technique

**DOI:** 10.1111/os.13645

**Published:** 2023-01-10

**Authors:** Hanru Ren, Lianghao Wu, Xu Zhang, Zheng Jian, Chengqing Yi

**Affiliations:** ^1^ Department of Orthopaedics, Shanghai Pudong Hospital Fudan University, Pudong Medical Center Shanghai China

**Keywords:** 3‐D fracture mapping technique, Clinical outcomes, New types, Proximal humerus fractures

## Abstract

**Objective:**

Fractures of different parts of the proximal humerus may lead to different postoperative functional deficits, but there are few studies on the morphology and related functions of the proximal humerus. The purpose of this study was to analyze the fracture pattern of the proximal humerus by the three‐dimensional (3‐D) fracture mapping technique and to further evaluate its clinical utility.

**Methods:**

Patients with proximal humeral fractures admitted to Pudong Hospital, Fudan University, from January 2018 to December 2020, were analyzed. Three surgeons divided the fractures into groups according to the 3‐D CT imaging technique and mapped the fractures on a 3‐D template according to the fracture line of each fracture. Finally, the humeral head inversion angle and the functional score were recorded in different fracture types.

**Results:**

A total of 312 cases of humeral fractures were included. Among them, there were 90 patients (28.8%) in the simple greater tuberosity + lesser tuberosity + medial cortex group, with typical fracture features of surgical neck fractures of the humerus + greater tuberosity fractures. Eighty‐seven patients (27.9%) in the greater tuberosity + isolated fragment lesser tuberosity + medial cortex group had typical “four‐part fractures.” There were 45 patients (14.4%) in the greater tuberosity + lesser tuberosity + medial isolated fragment group. Moreover, more patients in this group had medial comminution due to varus displacement of the femoral head. There were 66 patients (21.1%) in the isolated greater tuberosity group, 21 patients (6.7%) in the greater tuberosity + lesser tuberosity group, and three patients (1.0%) in the greater tuberosity + medial cortex group. In addition, the humeral head inversion angle and other statistical differences were observed in the greater tuberosity + lesser tuberosity + medial isolated fragment group.

**Conclusions:**

This morphological study helps to further identify the characteristics of proximal humerus fracture patterns, which may be closely related to different clinical outcomes. Further relevant studies are needed to verify the reliability of their clinical application and the potential value in surgical planning and postoperative functional rehabilitation.

## Graphical abstract

The study resulted in a summary of significant reproducible characteristics of six types involving proximal humerus fracture employing by 3‐D reconstruction and inferred patterns of Fracture Mappings. This morphological study helps to further identify and recognize the characteristics of fracture patterns in proximal humerus fractures.

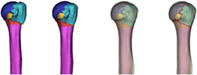

## Introduction

Fractures of the proximal humerus account for 5% of all fractures in the human body and are clinically common in older individuals with osteoporosis.^1^, ^2^ With the advent of an aging society, the number of patients with proximal humerus fractures is increasing annually. However, fracture displacement rates tend to be higher in proximal humerus fractures, with some studies reporting that at least 30% of proximal humerus fractures have a significant displacement.^3^ As a result, the function of the shoulder joint is often greatly affected.

The medial wall is an essential anatomical structure of the proximal humerus, and the importance of reconstructing the medial column support of the proximal humerus was suggested by Gardner *et al*.^4^ It has been suggested that preoperative comminuted fractures of the medial wall with poor repositioning increase the risk for postoperative complications such as inversion and collapse of the humeral head, failure of internal fixation, and screw cutting out of the articular surface.^5^, ^6^, ^7^, ^8^ Hence, providing reliable medial support while restoring the fracture is essential to reduce postoperative complications and ensure good functional recovery.^9^ The anterolateral structures of the proximal humerus mainly include the lesser tubercle and greater tuberosity of the humerus, which are the insertion points for the rotator cuff attachment. Fractures of the greater tuberosity may result in limited shoulder abduction, while comminuted fractures of the intertubercular sulcus may result in limited shoulder adduction. Consequently, there are variable possible postoperative complications of fractures in different regions of the proximal humerus, but the relevant type of article to address this issue is still lacking.

Therefore, the purpose of this study included the following points: (i) to perform morphological analysis of proximal humeral fractures by computed tomography (CT) reconstruction and fracture mapping techniques; (ii) to summarize common proximal humeral fracture patterns; and (iii) to analyze the differences in postoperative function, mobility, and complications between the different fracture types.

## Methods

### General Data

Patients diagnosed with proximal humeral fractures in our orthopedic database between January 2018 and December 2020 were retrospectively analyzed. The inclusion criteria were as follows: (i) fresh fractures with less than 2 weeks between injury and surgery; (ii) closed fractures; (iii) complete follow‐up for ≥12 months with reliable imaging data; and (iv) age > 18 years.

The exclusion criteria were as follows: (i) combined multiple injuries; (ii) pathological fractures; (iii) combined ipsilateral vascular and nerve injuries; and (iv) preexisting medical or surgical diseases affecting the shoulder function (frozen shoulder, rotator cuff injury, sequelae of cerebral hemorrhage).

The study was approved by the institutional ethics committee of Shanghai Pudong Hospital, which operates under the national and international standards (no. SWJWQN‐05), and was conducted following the latest version of the Declaration of Helsinki.

### Bone Block Area

Depending on the anatomical characteristics, we divided the structure of the proximal humerus into three fracture regions: the greater tuberosity, the lesser tuberosity, and the medial cortex. The greater tuberosity is the insertion point of the supraspinatus and infraspinatus, while the lesser tuberosity is the insertion point of the subscapularis. Therefore, injury to this area may affect abduction and adduction of the shoulder after surgery. The medial cortex plays a vital support role and is associated with postoperative complications such as re‐displacement. We, therefore, distinguished between anterior and medial fractures and classified fractures into different groups according to our regional grouping.

All fracture areas were grouped by two‐dimensional (2‐D) CT images and three‐dimensional (3‐D) reconstructed images in Picture Archiving and Communication System (PACS) and independently reviewed by three orthopedic surgeons experienced in treating proximal humeral fractures. If there was any disagreement, the resolution was reached through group discussion.

### Fracture Mapping

**Fig. 1:**
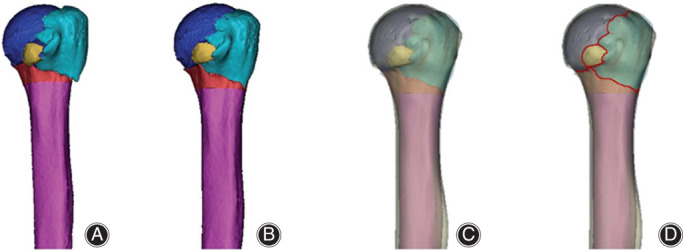
Three‐dimensional fracture‐mapping technique was used to prove the spatial morphology of proximal humerus fracture. CT data were used for reconstruction and virtually reduced fractures (A, B). Then, if necessary, other processes were performed to rotate, normalize, and flip the image to best match the 3‐dimensional template of the proximal humerus (C). Smooth curves are directly drawn on the surface of the 3‐D model to represent fracture lines (D)

A fracture mapping technique was used to describe the spatial pattern of fractures of the proximal humerus.^10^ Original Digital Imaging and Communications in Medicine (DICOM) files of selected CT scans were collected from the PACS database. Then, the DICOM data for all participants were uploaded to Mimics 21.0 software. Further CT data were used to reconstruct and reposition the fracture. Rotation, normalization, and flipping of the image were performed as needed to best match the 3‐D template of the proximal humerus (3‐Matic software; Materialise, Leuven, Belgium). Reference markers for alignment and standardization included the following parts: the medial and lateral bone contours of the proximal humerus, the greater tuberosity, the lesser tuberosity, the intertubercular sulcus, the humeral head, and the humeral shaft. Next, the reconstructed fragments were moved, rotated, and normalized to best fit the normal model of the proximal humerus. The fracture lines were represented by drawing smooth curves directly on the surface of the 3‐D model, and then the fracture lines were overlaid onto the 3‐D model to generate a spatial map. Finally, the fracture mappings were combined for each fracture type (Fig. 1).

### Imaging Findings and Function

**Fig. 2:**
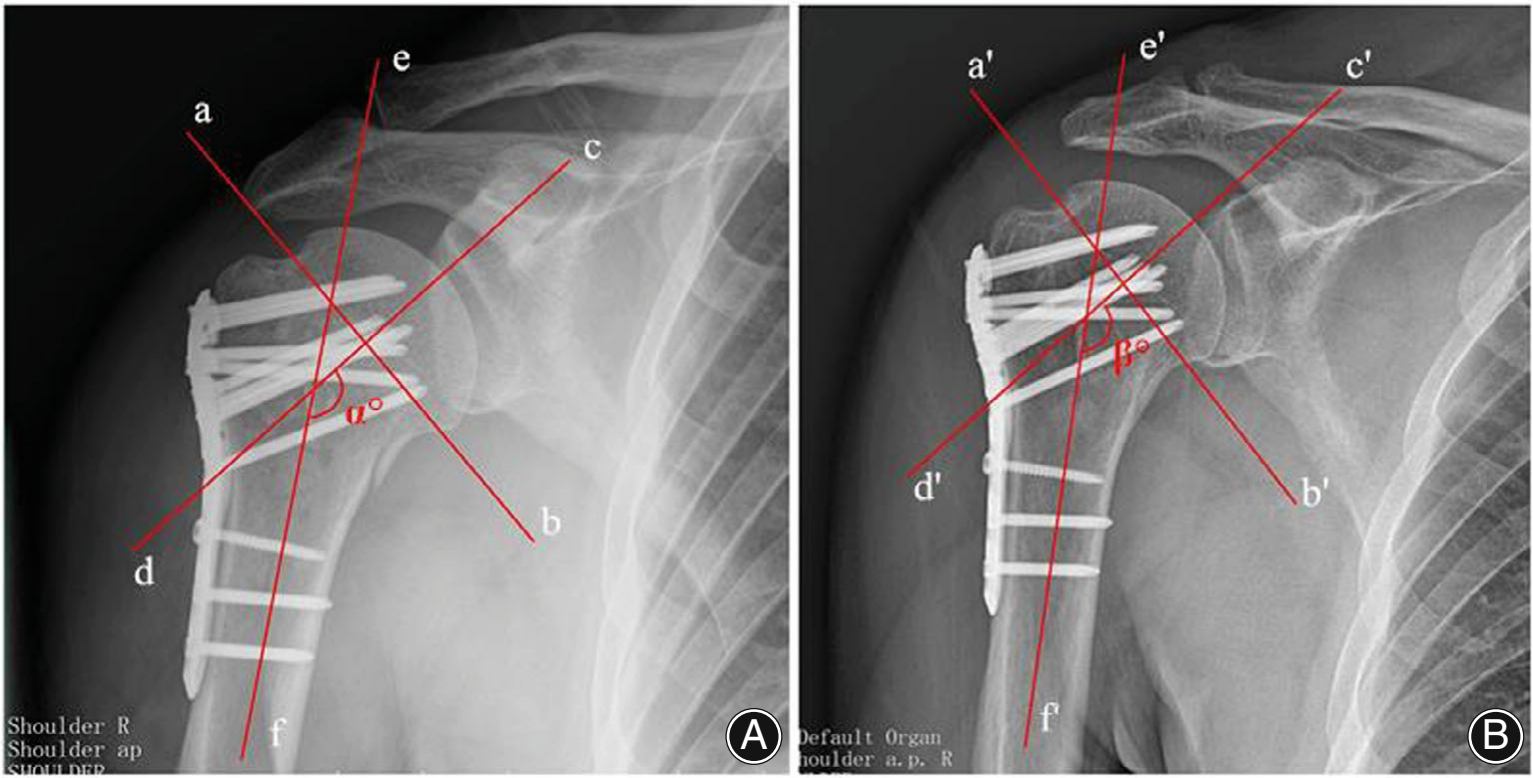
Measurement of humeral neck shaft angle changes. Immediate postoperative AP view (A). and last follow‐up (postoperative 12 months) AP view (B). the a, b (a′, b′) line was drawn from the superior to the inferior border of the articular surface. Then a perpendicular line was drawn(the c, d (c′, d′) line), through the center of the humeral head. The angle between the c, d (c′, d′) line and the line bisecting the humeral shaft (the e, f (e′, f′) line) was measured as the neck‐shaft angle.^11^ The humeral neck shaft angle change = α°‐β°

The postoperative stability was determined based on the degree of collapse of the humeral head. The degree of collapse of the humeral head was assessed by the change in the neck–shaft angle observed on anteroposterior radiographs of the shoulder joint. The change in the neck–shaft angle was measured immediately after the operation and 1 year after the operation (Fig. 2). We also collected each patient's Constant score to evaluate the postoperative recovery of the shoulder function.

### Statistical Analysis

The SPSS 22.0 statistical software (SSPS, Chicago, IL, USA) was used for all statistical analyses. Measured data were expressed as mean and standard deviation. Data were analyzed using one‐way analysis of variance (ANOVA), and comparisons between categorical data were performed using chi‐square tests and Fisher's exact tests. *P*‐values lower than 0.05 were considered statistically significant in all tests.

## Results

### General Characteristics of the Cases

We identified a total of 312 cases with proximal humeral fractures, 121 in males and 191 in females, with a mean age of 67.3 years. Table 1 shows the interclass correlation coefficient (ICC), 95% confidence interval (95% confidence interval [CI]) and descriptive level (*P* value) of angle measurement of three observers in 312 CT scans. A significant (*P* < 0.0001) intraobserver agreement existed for all three evaluators The consistency of observer 2 was the strongest (ICC = 0.881), and all observers showed high ICC values. In addition, we found only four different types of proximal humeral fractures and accordingly divided all of the cases into groups as follows: the isolated greater tuberosity injury group; the greater tuberosity + lesser tuberosity injury group; the greater tuberosity + medial cortex injury group; and the greater tuberosity + lesser tuberosity + medial cortex injury group.

**TABLE 1:** **Intraobserver** assessment using ICC

Observer	ICC	CI	*P* value
Observer 1	0.777	0.686–0.843	<0.0001
Observer 2	0.881	0.861–0.891	<0.0001
Observer 3	0.753	0.660–0.831	<0.0001

Abbreviations: CI, confidence interval; ICC, interclass correlation coefficient.

**Fig. 3:**
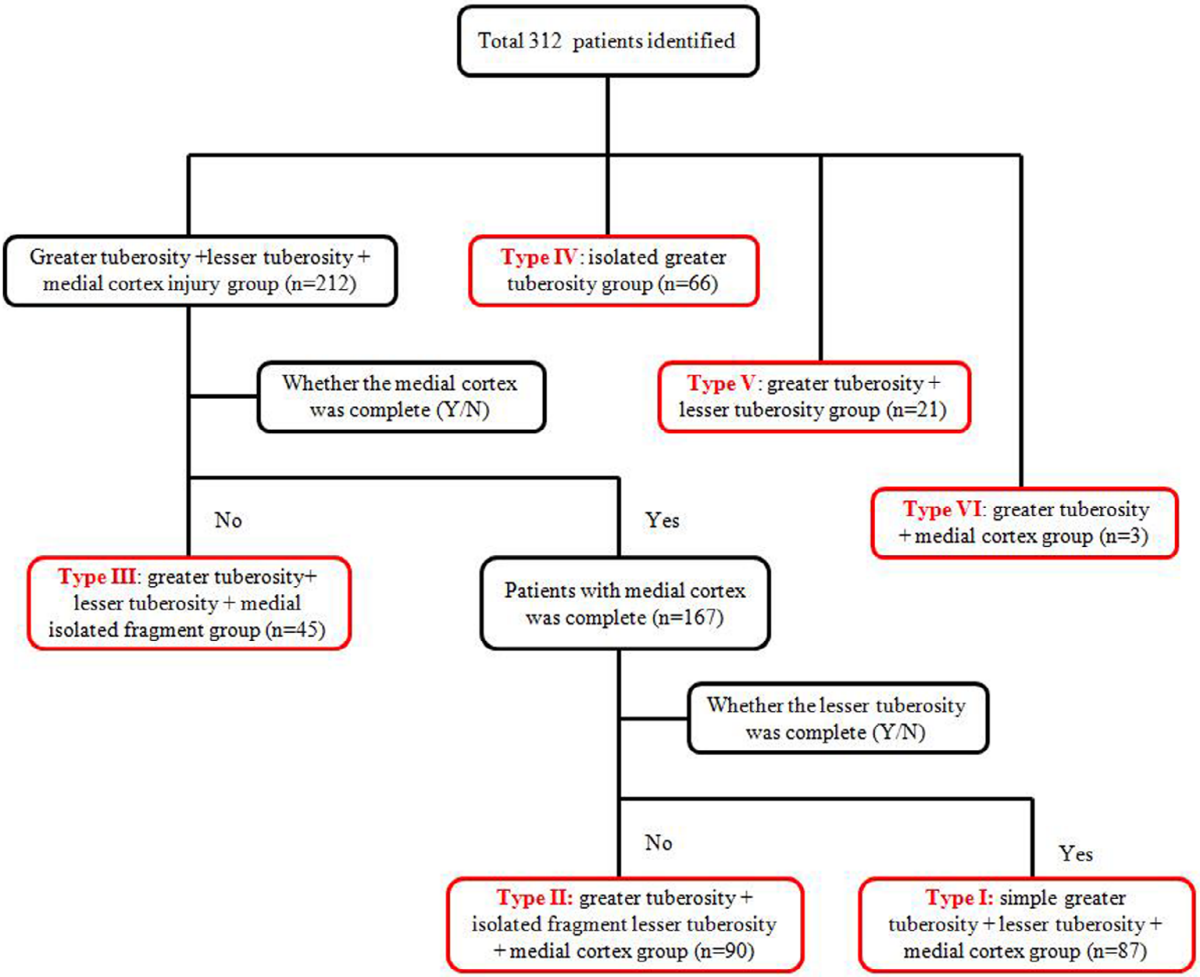
Flow diagram for analysis

**Fig. 4:**
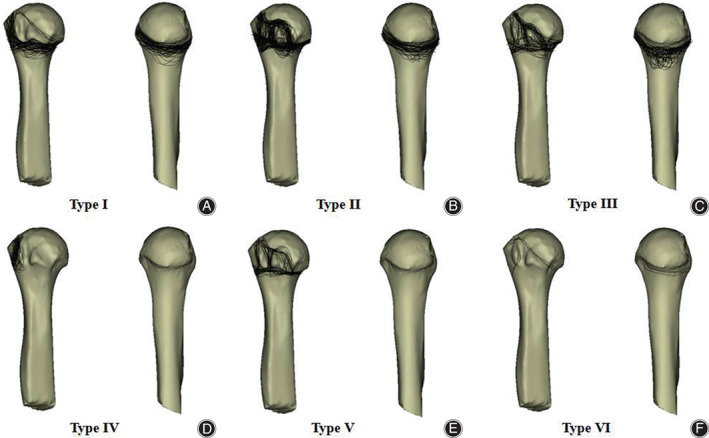
Representative views of the 3‐dimensional maps of the six proximal humeral fracture types. Fracture lines are depicted in black

We also found that all patients had concomitant fractures of the greater tuberosity. There were 212 cases (61.21%) in the greater tuberosity + lesser tuberosity + medial cortex injury group. We first defined the greater tuberosity + lesser tuberosity + medial isolated fragment group if the medial cortex was incomplete. Then, we defined the greater tuberosity + isolated fragment lesser tuberosity + medial cortex group if the lesser tuberosity had independent fragments. Finally, we analyzed six different types, namely 90 cases (28.8%) in the simple greater tuberosity + lesser tuberosity + medial cortex injury group, 87 cases (27.9%) in the greater tuberosity + isolated fragment lesser tuberosity + medial cortex group, 45 cases (14.4%) in the greater tuberosity + lesser tuberosity + medial isolated fragment group, 66 cases (21.1%) in the isolated greater tuberosity group, 21 cases (6.7%) in the greater tuberosity + lesser tuberosity group, and three cases in the greater tuberosity + medial cortex group (1.0%) (Figs. 3 and 4).

### Fracture Mapping

#### The Simple Greater Tuberosity + Lesser Tuberosity + Medial Cortex Group (Type I)

**Fig. 5:**
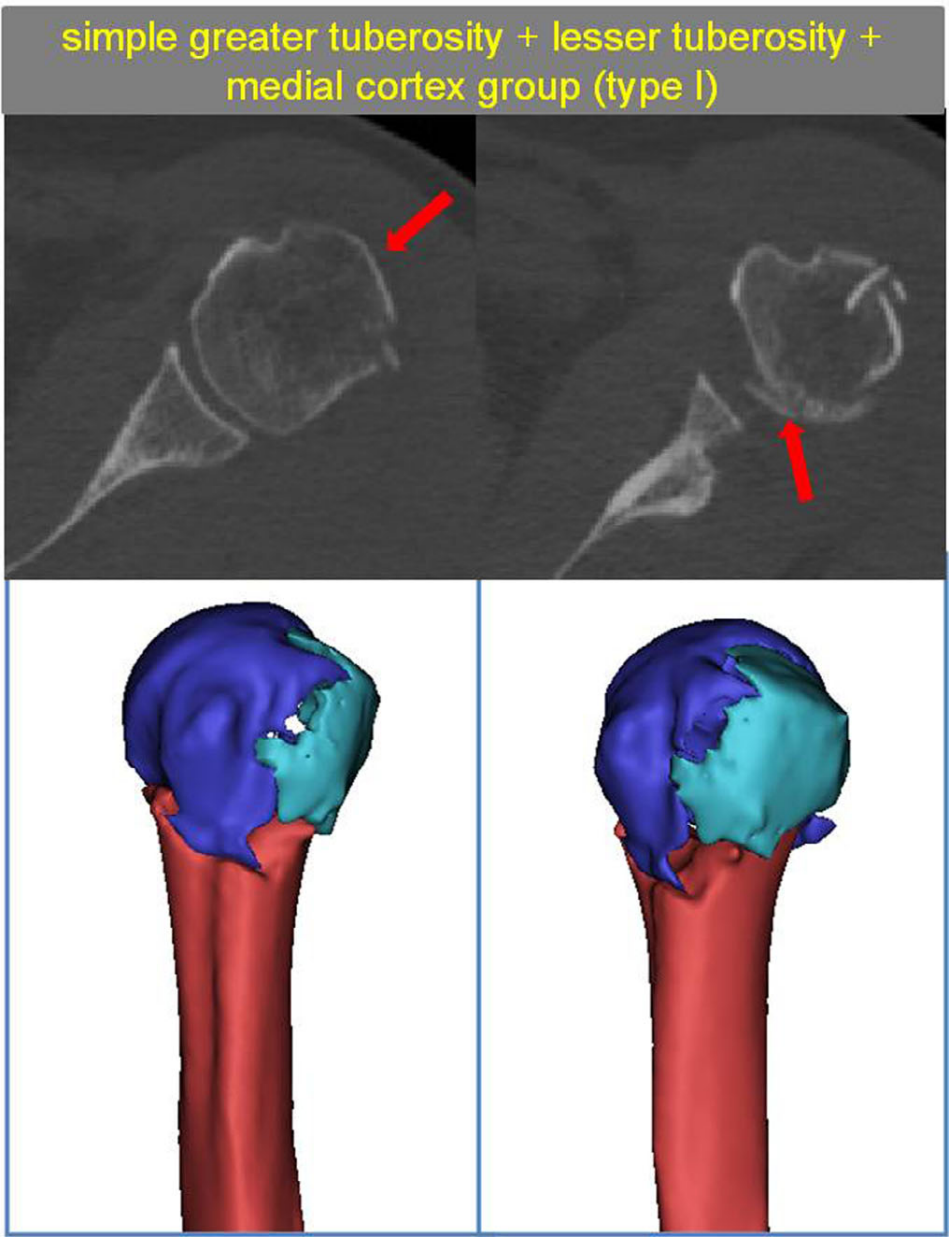
Type I with The simple greater tuberosity + lesser tuberosity + medial cortex group and the red arrow show greater tuberosity fragment

Including 90 patients, this group was the most common type of fracture pattern, and was typically characterized by a “three‐part fracture.” Surgical neck fractures of the humerus + greater tuberosity fractures were also commonly seen (87 cases), and such fractures divided the proximal humerus into three greater separate fragments, namely the greater tuberosity, the humeral head, and the humeral shaft. A few patients also had anatomical neck fractures + greater tuberosity fractures (Fig. 5).

#### The Greater Tuberosity + Isolated Fragment Lesser Tuberosity + Medial Cortex Group (Type II)

**Fig. 6:**
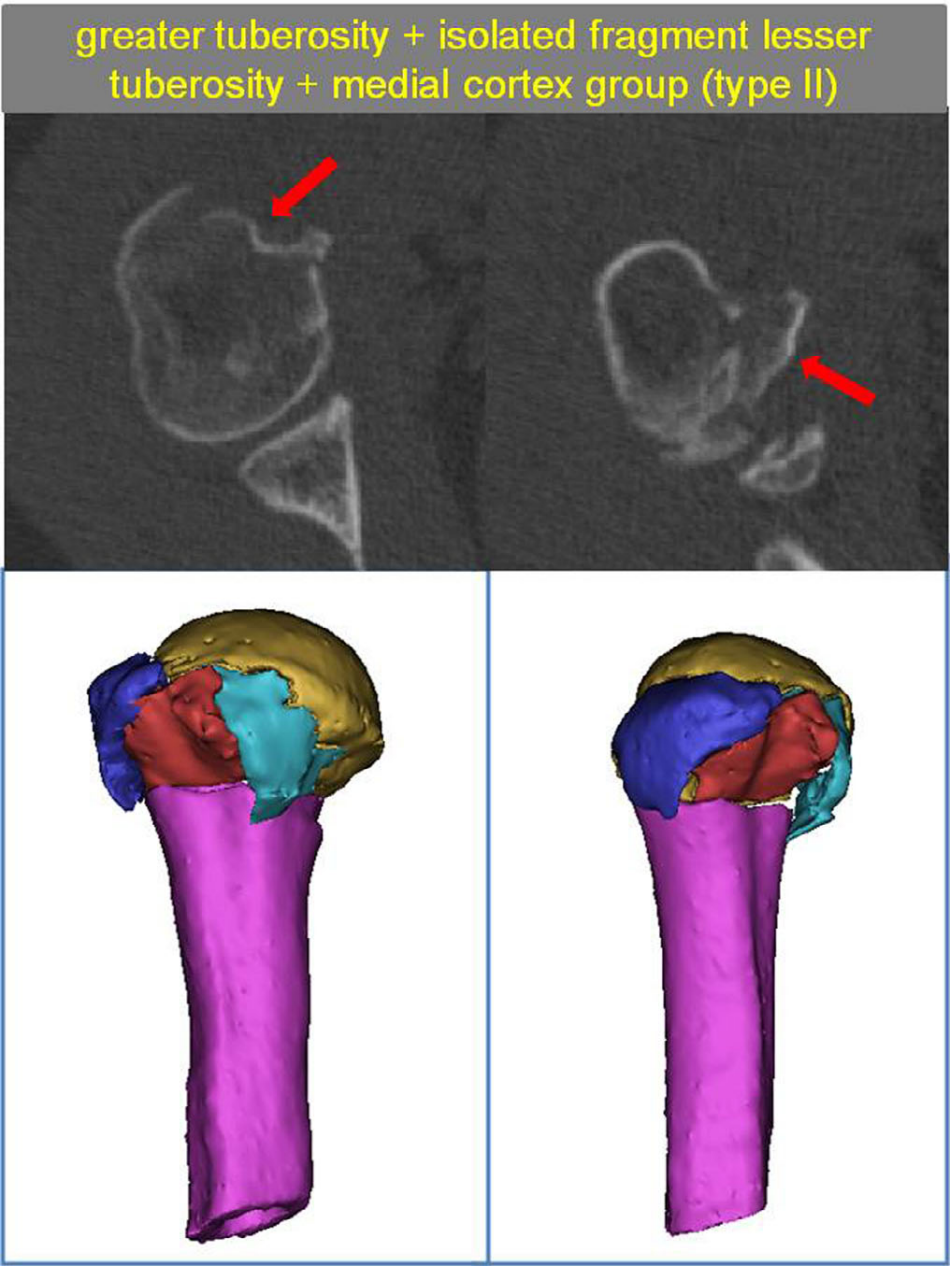
Type II with The greater tuberosity + isolated fragment lesser tuberosity + medial cortex group. The red arrow show isolated fragment lesser tuberosity and greater tuberosity fragment

This group comprised 87 patients and its most common fracture characteristic was a “four‐part fracture.” Namely, the fracture line divided the proximal humerus into four fragments (humeral head, greater tuberosity, lesser tuberosity, and humeral shaft). In addition, comminuted fractures of the greater tuberosity and the lesser tuberosity were seen in some of the patients (Fig. 6).

#### The Greater Tuberosity + Lesser Tuberosity + Medial Isolated Fragment Group (Type III)

With 45 patients included, this group had the most severe fractures. Specifically, all patients had comminuted fractures of the proximal humerus and displayed independent fragments of the lesser tuberosity, where the medial wall isolated fragment was defined as follows: a separate bone in the medial wall >1 cm in diameter; a comminuted fracture area in the medial wall.

**Fig. 7:**
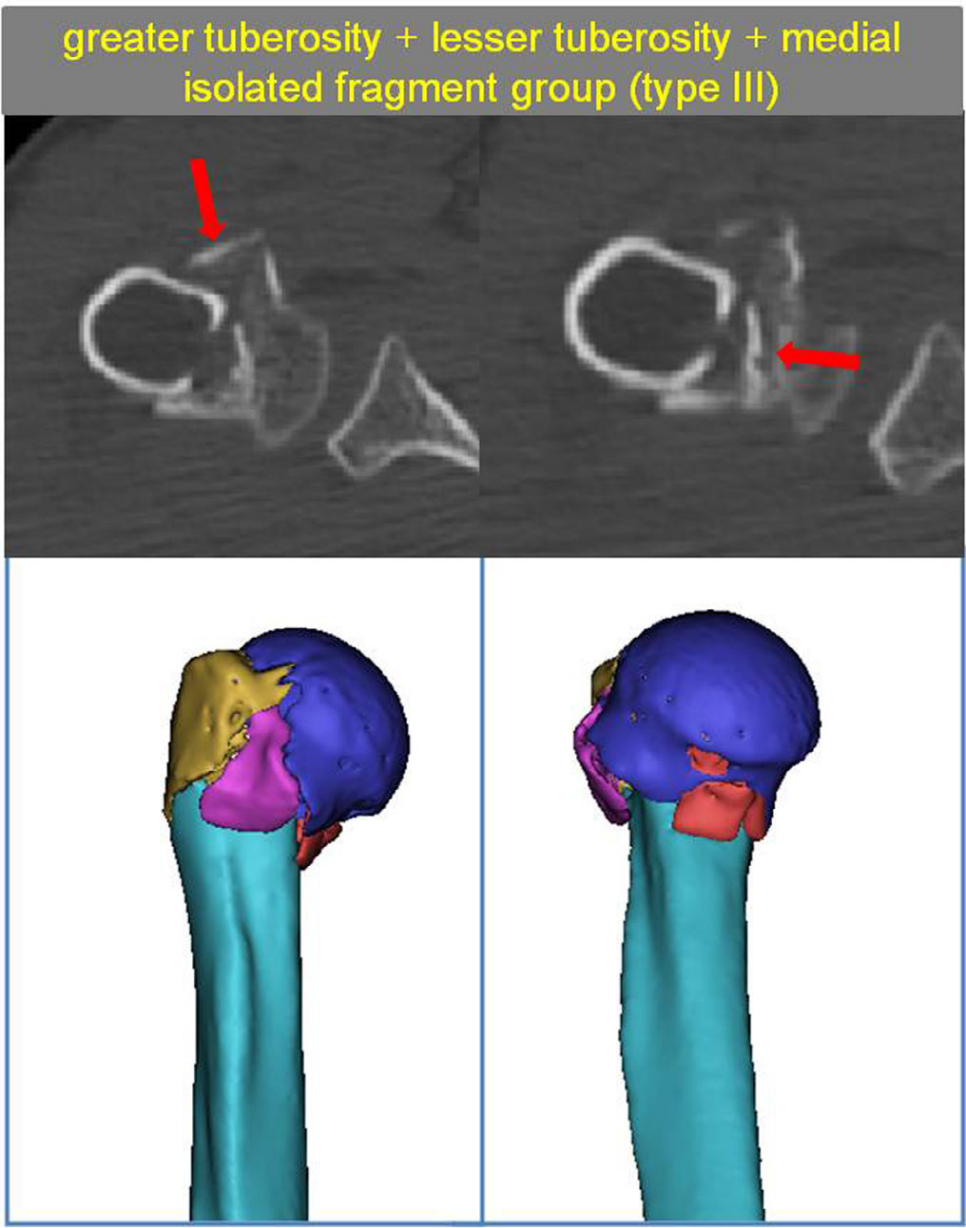
Type III with The greater tuberosity + lesser tuberosity + medial isolated fragment group. The red arrow show medial isolated fragment and lesser tuberosity fragment

In this group, 28 patients had severe inversion displacement of the femoral head, resulting in a comminuted fracture area in the lesser tuberosity and the medial wall. In the remaining 17 patients, a separate bone mass was observed medially. In the anterolateral region of the proximal humerus, patients with inversion displacement of the femoral head often had both a fracture of the anatomical neck and a fracture of the surgical neck (Fig. 7).

#### The Isolated Greater Tuberosity Group (Type IV)

**Fig. 8:**
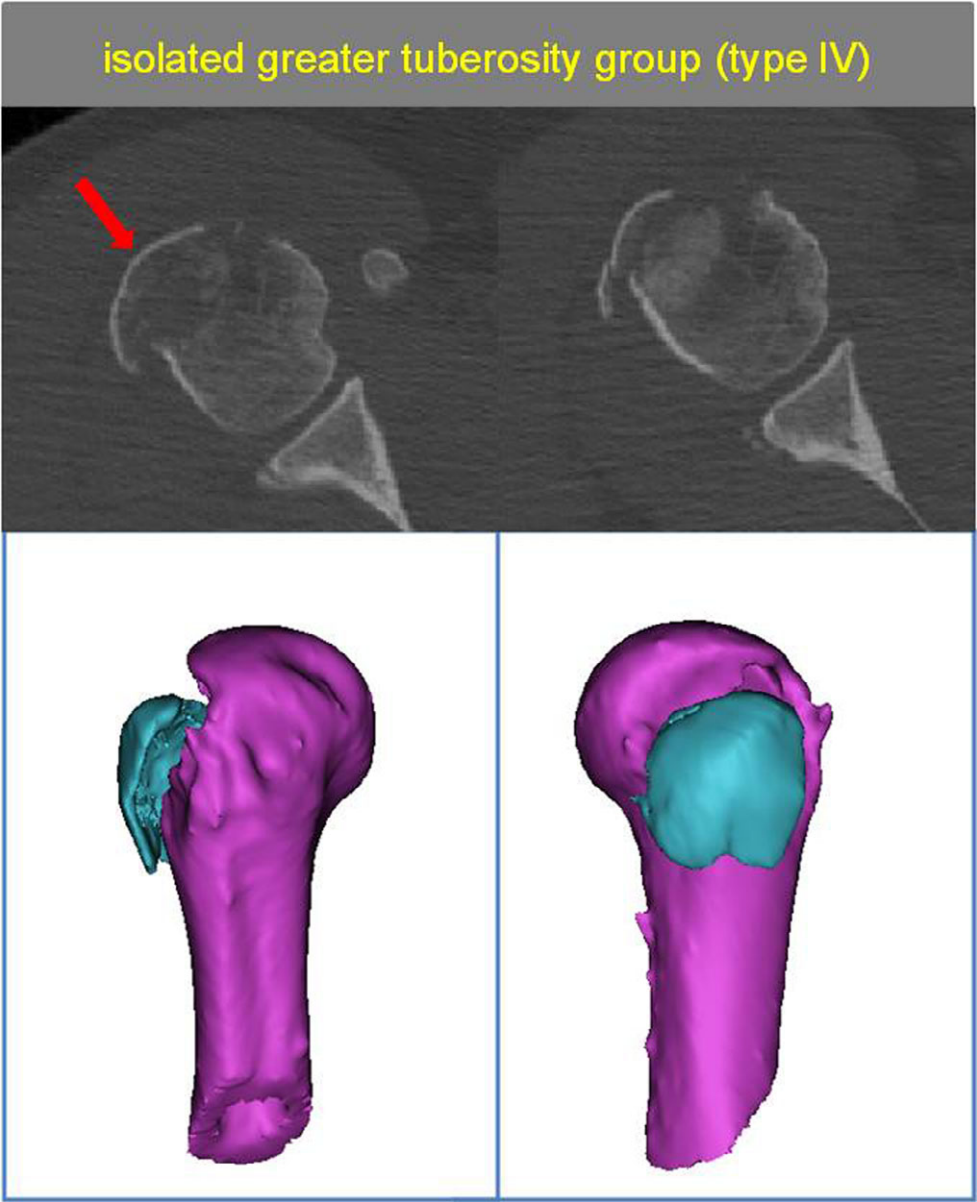
Type IV with The isolated greater tuberosity group. The red arrow show isolated greater tuberosity fragment

Comprising 66 patients, this group had a typical greater tuberosity fracture, where some patients had multiple fragments of the greater tuberosity most commonly with posterior medial displacement of the fragment (Fig. 8).

#### The Greater Tuberosity + Lesser Tuberosity Group (Type V)

**Fig. 9:**
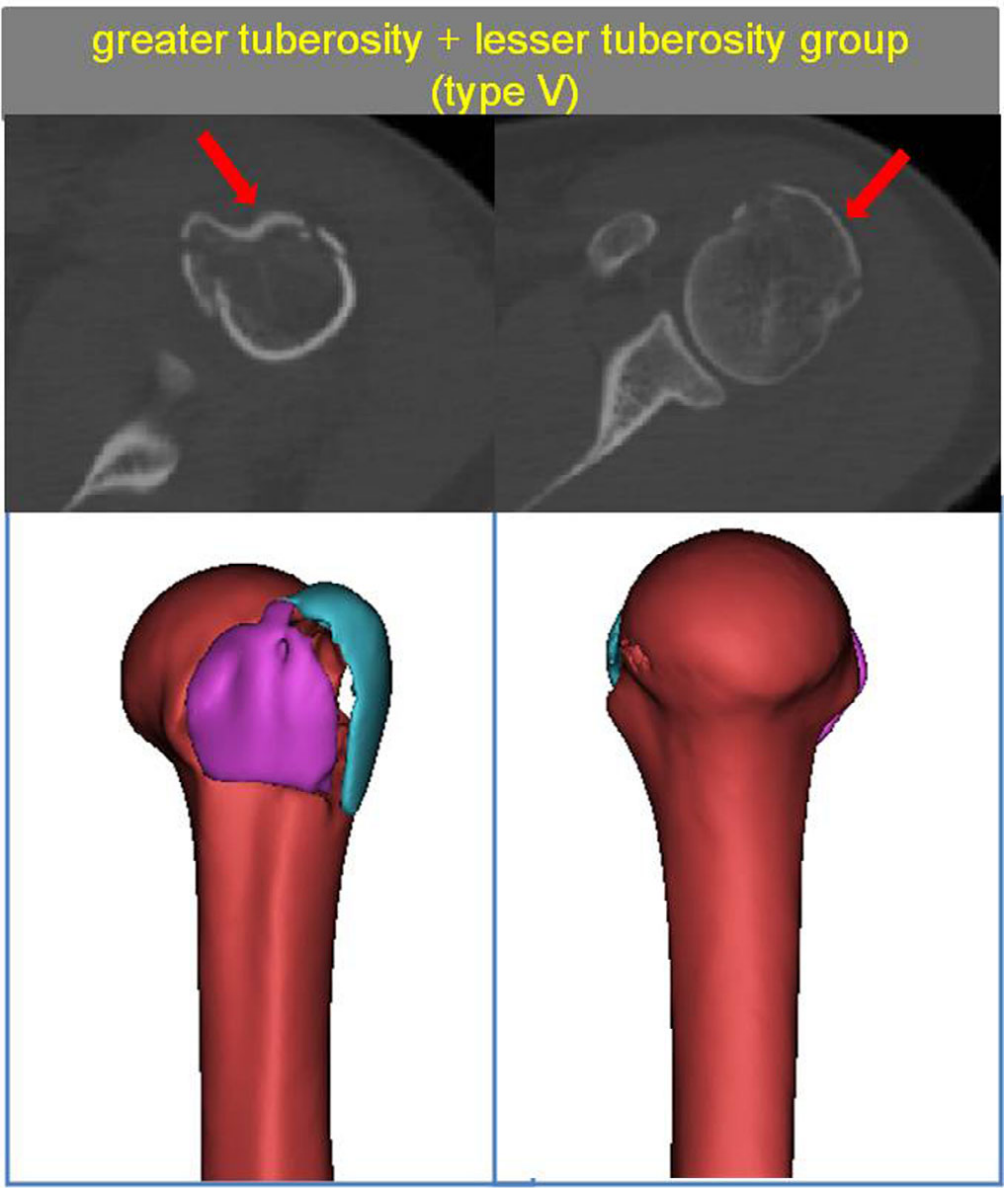
Type V with The greater tuberosity + lesser tuberosity group. The red arrow show greater tuberosity fragment and lesser tuberosity fragment

This group comprised 21 patients. The characteristics of anterolateral fracture in this group were similar to those in Type I and Type II proximal humerus fractures demonstrated in this study. A total of 13 patients had a fracture line extension to the intertubercular groove and the formation of a separate lesser tuberosity fragment. However, the humeral calcar of these patients was intact with no fracture line extension (Fig. 9).

#### The Greater Tuberosity + Medial Cortex Group (Type VI)

**Fig. 10:**
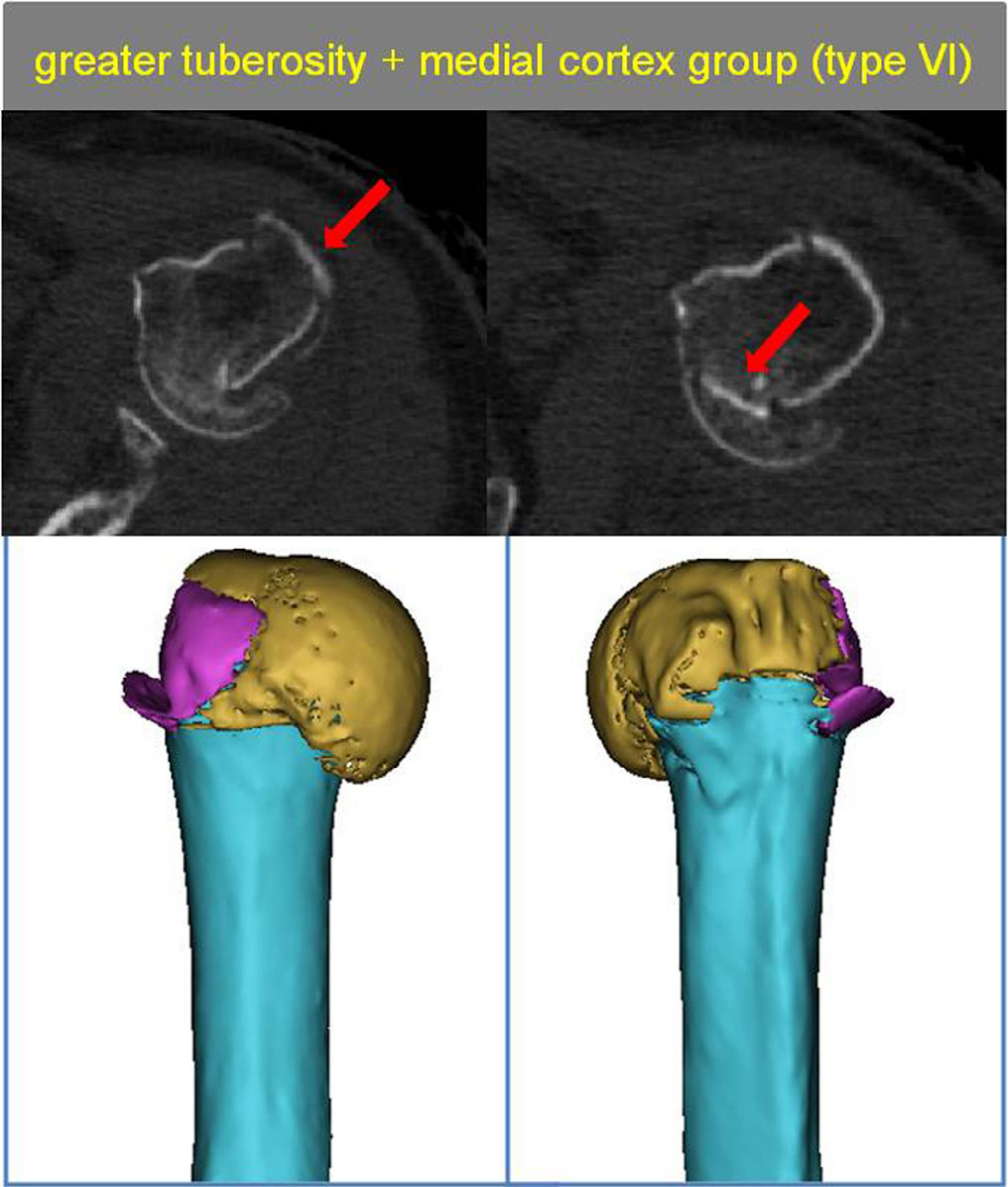
Type VI with The greater tuberosity + medial cortex group. The red arrow show greater tuberosity fragment and medial corte injury

Three patients had a fracture of the anatomical neck with a fracture of the greater tuberosity, in which the fracture line did not pass through the lesser tuberosity, so the lesser tuberosity remained attached to the humeral shaft (Fig. 10).

### Imaging Findings and Function

We measured the varus angle of the humeral head in all of the patients. There was a significant difference in humeral head varus angle between patients with Type III fractures and the other groups. In addition, the DASH score of patients with Type III fracture was the highest 28.75 ± 3.66, showing statistically significant differences between patients of different types. The results of patients' elevation function showed that the range of elevation of patients with Type II fracture was 123.7° ± 12.5°, and there were statistical differences between different groups. The range of activity of patients with Type III fracture in external rotation and internal rotation was the lowest 31.3° ± 8.5° and 38.7° ± 9.7°, respectively. Similarly, there were statistical differences between groups (Table 2).

**TABLE 2:** Postoperative humeral head varus angle, Constant Score, DASH Score, VAS Score and the ROM of shoulder of patients

Characteristic	I (n = 90)	II (n = 87)	III (n = 45)	IV (n = 66)	V (n = 21)	VI (n = 3)	F value	*P* value
Age (years)	62.3 ± 7.34	63.7 ± 5.23	67.6 ± 9.54	52.5 ± 7.78	55.6 ± 3.54		10.37	0.037*
Male/female	38/52	36/51	20/25	37/29	10/11	1/2		‐
Fracture type (Neer classification)								
2‐Part fracture	‐	‐	‐	66	‐	‐		‐
3‐Part fracture	90	‐	‐	‐	‐	3		‐
4‐Part fracture	‐	87	45	‐	21	‐		‐
Humeral head varus angle (°)	6.32 ± 3.04	7.23 ± 3.86	11.21 ± 4.57	1.14 ± 0.54	1.17 ± 0.91	‐	21.22	0.007 *
Constant Score	83.14 ± 7.31	78.51 ± 7.33	76.17 ± 8.21	90.12 ± 3.55	87.53 ± 5.95	‐	20.76	0.009 *
DASH Score	20.71 ± 4.97	25.45 ± 3.24	28.75 ± 3.66	15.57 ± 7.14	18.38 ± 2.82		18.54	0.013 *
VAS Score	1.31 ± 0.21	1.30 ± 0.37	1.77 ± 1.20	0.72 ± 0.16	0.31 ± 0.22	‐	1.54	0.088
Elevation (°)	135.2 ± 7.2	123.7 ± 12.5	127.7 ± 11.8	143.8 ± 8.2	137.3 ± 6.4	‐	12.57	0.033 *
External rotation (°)	37.3 ± 6.2	35.7 ± 6.8	31.3 ± 8.5	55.9 ± 8.9	48.6 ± 3.7		13.78	0.029 *
Internal rotation (°)	48.6 ± 7.1	41.8 ± 8.3	38.7 ± 9.7	61.9 ± 6.9	57.6 ± 4.8	‐	15.67	0.021 *

* *P* < 0.05 was considered significant.

## Discussion

In this study, we reconstructed 312 proximal humerus fracture models from CT scans and performed a macroscopic analysis of proximal humerus fracture morphology using the fracture mapping technique.^12^ Analysis of the proximal humerus fracture morphology allowed us to further improve our understanding of this fracture type. We divided proximal humerus fractures into six distinct groups by summarizing the most common fracture morphological characteristics in different types of fractures.

### Fracture Mapping Completes the Morphological Analysis of Proximal Humerus Fractures

The fracture mapping technique allows a more 3‐D presentation of the fracture line and fracture morphology of the proximal humerus fractures. By analyzing the morphology of different areas of the proximal humerus fractures, we could gain further insight into proximal humerus fracture injuries.^13^, ^14^ We believe that in proximal humerus fracture injuries, medial, anterior (lesser tuberosity area), and lateral (greater tuberosity area) injuries represent different injury patterns, which produce different postoperative changes in the shoulder function and range of motion.

### Fracture Mapping and 3‐D Typing of Proximal Humerus Fractures

The 4‐part fracture classification based on X‐rays is still the system that is most frequently used to categorize proximal humeral fractures, but more and more scholars are beginning to pay attention to the classification system based on CT scans.^15^, ^16^, ^17^ Edelson *et al*. proposed the Edelson fractal system based on CT 3‐D reconstruction and proposed the “shield” fracture concept.^18^ However, we believe that good fracture typing should be able to diagnose fractures, guide treatment accurately, and predict the prognosis of fractures. Simultaneous sustained reproducibility and ease of use are also important, indicating that fracture typing needs to be easily understood and applied by clinicians while also providing almost consistent results across clinicians.

In addition, we believe that the integrity of the medial structure plays an essential biomechanical role in ensuring the stability of the proximal humerus; hence, the absence of the medial support is an important cause of the humeral head collapse, inversion deformity, screw cutout, and failure of internal fixation.^19^ In recent years, more and more scholars have discovered the importance of the humeral calcar.^20^ Moreover, several scholars have recently proposed approaches to reconstruct the medial column.^21^, ^22^ However, no fracture typology is associated with the absence of the medial column. The lack of the medial wall support in our study was divided into separate types, corresponding to particular fracture types of the proximal humerus, providing a more precise classification of the proximal humerus region. We also elucidated the relevance of our typing to clinical management by retrospectively analyzing the imaging findings and function of patients with different fracture types. We believe that the inferred pattern of the fracture mapping can provide a new spatial perspective on the fracture pattern and morphology of the anteromedial bone mass of the proximal humerus fracture, ultimately revealing the clinical features of the different underlying patterns.

In our study, all proximal humerus fractures included fractures of the greater tuberosity, which may be related to the insufficient number of cases, but the real reason for the high incidence of greater tuberosity fractures remains unclear. It is believed that part of the cases may have developed due to the direct force on the greater tuberosity. In addition, the pulling effect of the supraspinatus on the greater tuberosity during trauma and the protective effect of the subscapularis on the anteromedial region are equally responsible for the greater tuberosity being more prone to fracture.

Through the use of fracture mapping technology, we classified proximal humeral fractures into six types during the investigation. First, compared to conventional classification methods, the classification based on CT 3D reconstruction has a superior performance in repeatability, which is highly consistent with the results shown in Table 1 by three separate observers. Second, despite the fact that we have long focused on the impact that humeral calcar fractures have on patients' ability to function following surgery, traditional classifications have failed to adequately address the damage to the humeral calcar and whether a comminuted fracture of the humeral calcar will ultimately cause the humeral head to collapse, the humeral neck to shorten, and the failure of internal fixation after surgery. Regarding our classification, we first suggested the idea of an independent fragment of the humerus calcar, which is present in patients with Type III fractures' medial wall of the humerus. After surgery, there might still be a partial loss of the medial cortex, which would lead to postoperative instability, according to our theory. Additionally, we discover that patients with Type III fractures were more likely to develop humeral head collapse and varus deformity over the course of their treatment. In addition, patients with Type III fractures demonstrated a poor functional status following fracture, with statistically significant disparities between their Constant and DASH functional ratings and those of other groups.

Therefore, if we encounter a patient with a Type III proximal humeral fracture in the future, we will focus on the following issues, including medial wall reconstruction, whether the biomechanics of humeral calcar screw is sufficient, whether additional fixation and joint bone grafting are required, and so on. This will help the patient's postoperative function return more quickly. Results from postoperative functional exercises demonstrate that patients with IV and V fractures have considerably improved internal and exterior rotation function compared to patients with other types of fractures. We hypothesize that this is because Type IV and V fracture patients' tubercles are intact and their injuries to the internal and external rotation tendons' insertion points are relatively minor, leading to greater postoperative function. We are aware that the shoulder joint is a somewhat unique joint. Long‐term braking may result in postoperative shoulder joint adhesion and joint stiffness, which will hinder the healing of patients; therefore, we must determine which patients are suitable for early exercise. Research has led us to assume that patients with Type I, IV, and V fractures have stable internal fixation following surgery and are able to perform their duties earlier, hence lowering the risk of postoperative adhesion and shoulder joint stiffness. Patients with Type II and III, however, need to worry about a more careful rehabilitation exercise.

### Limitations and Strengths

There are some limitations in the study. First, the study was retrospective and lacked an assessment of shoulder mobility in patients. We suppose that the entire pathophysiology of the fracture is related to the muscle and ligament attachment points at the fracture site. Therefore, injuries in different areas may lead to different limitations in the range of motion of the shoulder joint, so we need to collect more data for further refinement. In addition, some patients with simple lesser tuberosity fractures and simple greater tuberosity fractures opted for conservative treatment. They were not accounted for in this study, which may have led to a bias in the proportion of different fracture types. More prospective studies are needed to verify the validity of this new classification system. Finally, this study has not yet tested intra‐ and inter‐observer consistency.

The strength of this study involves the innovative use of fracture mapping technology to analyze the morphology of proximal humerus fractures and summarize six groups of fracture types with typical morphology. This CT‐based grouping is not only more convenient, but also there are typical differences between different types of patients after surgery, which is convenient for guiding future clinical work.

### Conclusions

We summarized significantly reproducible characteristics of six types of proximal humerus fractures employing 3‐D reconstruction and inferred patterns of fracture mappings. This morphological study helps to further identify and recognize the characteristics of fracture patterns in proximal humerus fractures. These different fracture patterns may be closely associated with different clinical prognoses. Undoubtedly, further studies are needed to verify its clinical application's reliability and potential value in surgical planning and postoperative functional rehabilitation.

## Ethics Approval and Consent to Participate

Ethics approval and consent to participate was obtained at the “Ethics Committee Shanghai Pudong Hospital” to perform this retrospective review, and the number is (WZ‐005). In addition, all patients signed a informed consent.

## Authors' Contributions

HR and LW designed the study, collected, analyzed and interpreted the data, and wrote the paper. CY designed the study, analyzed and interpreted the data. XZ collected and interpreted the data. ZJ is been involved in drafting the manuscript. All authors read and approved the final manuscript.

## Data Availability

The datasets used and analyzed during the current study are available from the corresponding author on reasonable request.
